# Smarter Traffic Prediction Using Big Data, In-Memory Computing, Deep Learning and GPUs

**DOI:** 10.3390/s19092206

**Published:** 2019-05-13

**Authors:** Muhammad Aqib, Rashid Mehmood, Ahmed Alzahrani, Iyad Katib, Aiiad Albeshri, Saleh M. Altowaijri

**Affiliations:** 1 Department of Computer Science, FCIT, King Abdulaziz University, Jeddah 21589, Kingdom of Saudi Arabia; mpervez@stu.kau.edu.sa (M.A.); asalzahrani@kau.edu.sa (A.A.); iakatib@kau.edu.sa (I.K.); aaalbeshri@kau.edu.sa (A.A.); 2 High Performance Computing Center, King Abdulaziz University, Jeddah 21589, Kingdom of Saudi Arabia; 3 Faculty of Computing and Information Technology, Northern Border University, Rafha 91911, Kingdom of Saudi Arabia; Saltowaijri@nbu.edu.sa

**Keywords:** road traffic prediction, smart cities, deep learning, TensorFlow, graphics processing units (GPUs), convolution neural networks, in-memory computing, big data, smart transportation

## Abstract

Road transportation is the backbone of modern economies, albeit it annually costs 1.25 million deaths and trillions of dollars to the global economy, and damages public health and the environment. Deep learning is among the leading-edge methods used for transportation-related predictions, however, the existing works are in their infancy, and fall short in multiple respects, including the use of datasets with limited sizes and scopes, and insufficient depth of the deep learning studies. This paper provides a novel and comprehensive approach toward large-scale, faster, and real-time traffic prediction by bringing four complementary cutting-edge technologies together: big data, deep learning, in-memory computing, and Graphics Processing Units (GPUs). We trained deep networks using over 11 years of data provided by the California Department of Transportation (Caltrans), the largest dataset that has been used in deep learning studies. Several combinations of the input attributes of the data along with various network configurations of the deep learning models were investigated for training and prediction purposes. The use of the pre-trained model for real-time prediction was explored. The paper contributes novel deep learning models, algorithms, implementation, analytics methodology, and software tool for smart cities, big data, high performance computing, and their convergence.

## 1. Introduction

Smart cities appear as “the next stage of urbanization, subsequent to the knowledge-based economy, digital economy, and intelligent economy” [1]. Smart cities aim to “not only exploit physical and digital infrastructure for urban development but also the intellectual and social capital as its core ingredient for urbanization” [1]. Smart cities are driven by, or involve, integration of multiple city systems, such as transport, healthcare, and operations, and hence are considered a major driver for the transformation of many industries. Smart society is an extension of the smart cities concept, “a digitally-enabled, knowledge-based society, aware of and working towards social, environmental and economic sustainability” [1].

Road transportation is the backbone of modern economies, albeit it annually costs 1.25 million deaths and another 20–50 million injuries to people [2]. This equals daily a shocking 3400 deaths and 50,000–137,000 injuries to people. Moreover, trillions of dollars of the global economy are lost due to road congestion in addition to the congestion causing air pollution that damages public health and the environment [3].

INRIX Research conducted the biggest study on congestion costs based on the data acquired by 300 million vehicles and devices from 1360 cities in 38 countries during 2017 [4]. They revealed that Los Angeles was the worst congested city globally where drivers spent 102 peak hours in congestion, equaling an average of 12% of their total drive time, the congestion in the city costing $19.2 billion to the drivers and US economy. The cost of congestion in New York was the highest at $33.7 billion for any single city in the world. The total congestion cost across the US, UK, and Germany was about $461 billion. The cost of congestion to the United States economy, alone, exceeds $305 billion (see [4,5]). Texas Transportation reports that 2.9 billion gallons of fuel was wasted in the USA alone during 2012 due to traffic congestion [6], and this causes, in addition to the economic losses, damage to the environment and public health. It is reported [7] that 40% of the road congestion is caused due to the bottlenecks on the road networks, 25% is because of the traffic crashes and other incidents, 15% is caused due to the bad weather, and the remaining 20% includes other causes such as roadworks and poor traffic signal control. Better insights into the causes of road congestion, and its management, are of vital significance to avoid or minimize loss to public health, deaths and injuries, and other socio-economic losses and environmental damages.

Many road traffic modeling, analysis, and prediction methods have been developed to understand the causes of road traffic congestion, and to prevent and manage road congestion. The forecasting or prediction of road traffic characteristics, such as speed, flow and occupancy, allows planning new road networks, modifications to existing road networks, or developing new traffic control strategies. Real-time traffic prediction allows dynamic control of road traffic using traffic signal controls, variable lane control, variable message signs (VMS), and other methods. Simulations and modeling methods have been widely used in the past for road traffic management and congestion prevention (see, e.g., [8,9,10,11,12,13,14,15,16,17,18]). In recent years, statistical methods, artificial intelligence (AI) and data mining techniques are increasingly being used to analyze road traffic data and predict future traffic characteristics. These include the Autoregressive Integrated Moving Average (ARIMA) [19], and its variants Seasonal Autoregressive Integrated Moving Average (SARIMA) [20], and KARIMA (Kohonen Maps with ARIMA) [21]. Support vector machines (SVM) [22] are also popular for the analysis of transportation problems. Deep learning is being used recently for traffic prediction purpose [23,24,25,26,27,28,29,30]. Deep learning is a branch of machine learning that uses hierarchical architectures to learn high-level abstractions in the data [31].

The focus of this work is on the use of deep learning for road traffic prediction. The existing literature on the use of deep learning for road traffic prediction is in its infancy and falls short in multiple respects. Firstly, the datasets that have been used are relatively small in terms of the time duration of the road traffic data. Secondly, some of the deep learning works use data from cameras installed by the researchers and, although useful and complementary, this renders such datasets limited in their scopes as compared to the data systematically collected by transportation authorities. Thirdly, the existing deep learning works have not reported in-depth analysis of the configurations of deep learning networks. Therefore, further work is needed on the use of deep learning for road traffic modeling and prediction.

Relatedly, the last few decades have seen an increasing surge in the technological advancements. The penetration of these technologies to all spheres of everyday life has given rise to the smart infrastructure developments; smart transportation infrastructure is at the forefront of these developments [1,32,33,34,35,36]. The use of GPS devices and mobile signals to collect vehicle location and congestion data [37]; the use of big data [38,39,40] and high performance computing (HPC) [38,40,41,42] technologies; mobile, cloud and fog computing [37,43,44,45,46]; image processing and artificial intelligence (AI) for traffic analysis [47]; urban logistics prototyping [48]; vehicular ad hoc networks [44,49,50,51,52]; autonomous driving [47]; autonomic transportation systems [53,54,55]; and the use of social media for traffic event detection [56,57,58] are a few examples. There is a need for innovative uses of the cutting-edge technologies in transportation.

This paper addresses the challenges of road traffic prediction by bringing four complementary cutting-edge technologies together: big data, deep learning, in-memory computing, and high performance computing (Graphics Processing Units (GPUs)). The approach presented in this paper provides a novel and comprehensive approach toward large-scale, faster, and real-time road traffic prediction. The road traffic characteristics that we predict are flow, speed, and occupancy. Big data refers to the “emerging technologies that are designed to extract value from data having four Vs characteristics; volume, variety, velocity and veracity” [59]. GPUs provide massively parallel computing power to speed up computations. Big data leverages distributed and high performance computing (HPC) technologies, such as GPUs, to manage and analyze data. Big data and HPC technologies are converging to address their individual limitations and exploit their synergies [60,61]. In-memory computing allows faster analysis of data by the use of random access memories (RAMs) as opposed to the secondary memories. We used Convolutional Neural Networks (CNNs) in our deep learning model.

The dataset we used is provided publicly by the California Department of Transportation (Caltrans) Performance Measurement System (PeMS) [62]. The road traffic dataset provides five-minute interval traffic data on the freeways. It includes vehicle flow, speed, occupancy, the ID of the Vehicle Detector Station (VDS), and other data. The dataset is used for the training of deep convolution neural networks. We analyzed over 11 years of PeMS road traffic data from 2006 to 2017 collected from the 26 VDSs on a selected chunk of a big corridor I5-N in California. To the best of our knowledge, this is the largest data, in terms of the time duration, that has been used in a deep learning based study. Big data veracity issues have been discussed in detail and methods to address the incompleteness and errors in data have been described.

Several combinations of the input attributes of the data along with various network configurations of the deep learning models are investigated for the training and prediction purposes. Different configuration sets of the deep learning networks have been executed multiple times, where the batch sizes and the number of epochs have been varied with different combinations, and each combination has been executed multiple times. These multiple configurations show consistency of the accuracy of the results. The training of a deep model is a compute intensive job, particularly when the size of the dataset is large. The use of GPUs provides a speedy deep learning training process, and we verified this by comparing the execution time performance of the training process on GPUs with CPUs. Moreover, we explored the possibility of real-time prediction by saving the pre-trained deep learning models for traffic prediction using the complete 11 years of data, and subsequently using it on smaller datasets for near real-time traffic predictions using GPUs. This is a first step towards the real-time prediction of road traffic and will be further explored in our future work. For the accuracy evaluation of our deep prediction models, we used three well-known evaluation metrics: mean absolute error (MAE), mean absolute percentage error (MAPE), and root mean squared error (RMSE). Additionally, we have provided the comparison of actual and predicted values of the road traffic characteristics. The results demonstrate remarkably high accuracy.

The paper contributes novel deep learning models, algorithms, implementation and an analytics methodology, and a software tool for smart cities, big data, HPC, and their convergence. The paper also serves as a preliminary investigation into the convergence of big data and higher performance computing [60] using the transportation case study. These convergence issues will be further explored in the future with the aim of devising novel multidisciplinary technologies for transportation and other sectors.

The rest of this paper is organized as follows. Section 2 reviews the work done by others in the area of traffic behavior prediction. The details about our methodology and the deep learning model are given in Section 3. The input dataset and pre-processing details are also described in this section. Section 4 discusses the results. The proposed approach for the near real-time prediction of road traffic is discussed in Section 5. Finally, we conclude in Section 6 and give directions for future work.

## 2. Literature Review

A great deal of works have been done on traffic modeling, analysis, and prediction, and in the broader area of transportation. Some of these works have already been mentioned and discussed in Section 1. Our focus in this paper is on the road traffic characteristics prediction using deep learning approaches, and, hence, in the rest of this section, we review the notable works relevant to our main focus area in this paper.

A deep learning approach to predict traffic flow for short intervals on road networks is proposed in [26]. A traffic prediction method based on long short-term memory (LSTM) was used by the authors for prediction purpose. An origin destination correlation (ODC) matrix is used as input to the training algorithm. The dataset used for this process is retrieved from the Beijing Traffic Management Bureau and is collected from more than 500 observation stations or sensors containing around 26 million records. Five-minute interval data from 1 January 2015 to 30 June 2015 are collected where the data for first five months are used for training and the rest of the data were are for testing purposes. For evaluation of proposed model, mean absolute error (MAE), mean square error (MSE), and mean relative error (MRE) are calculated. Input data are used to predict the flow in 15-, 30-, 45-, and 60-min time intervals. The authors selected three observation points with high, medium, and low flow rates to compare the actual flow and predicted flow values on those observation points. MRE values for 15-min interval flow prediction reported in this work are 6.41%, 6.05%, and 6.21%. They compared the result with the other approaches including RNN, ARIMA, SVM, RBF, etc. and concluded that for time interval shorter than 15 min, RNN is relatively accurate, but, with big time intervals, error increases. Overall, it performs better than other older machine learning models. Therefore, it is concluded that LSTM is an appropriate choice for long time intervals.

Another traffic flow prediction approach has been proposed in [23] that uses autoencoders for training, testing and to make predictions. The model is named as stacked autoencoder (SAE) model. Data for this purpose are also collected from PeMS [62]. They used weekdays (Monday–Friday) data for first three months of 2013 giving vehicles flow on a highway on five-minute interval basis. Data for first two months are used for training and remaining one-month data are used for the testing. By using five-minute interval data, the authors predicted the aggregate flow for 15-, 30-, 45-, and 60-min intervals in this model. Three months of data for all the highways available in the system are used in this model. Although this is a large amount of data that requires gigabytes of storage capacity, the key point to mention is that data from one highway can only be used to predict the flow on that particular highway. Thus, if they used each highway’s data to predict the flow of vehicles on each highway, then it means that they executed the proposed model with different small datasets (few MBs each) to predict the flow values for each highway. Therefore, no big data technology is used to store or process the input datasets. Support vector machines (SVM) have been used for comparison purpose by using three performance metrics, i.e., mean absolute error (MAE), mean relative error (MRE), and root mean squared error (RMSE). Flow values for four time-intervals (15-, 30-, 45-, and 60-min) are predicted and the authors claimed the average accuracy for all four intervals of 93% obtained by using MRE% values (i.e., 1 − MRE%).

In another work [27], a deep learning approach using CNN and LSTM is proposed to predict the traffic flow and congestion on highways. First, CNN is applied on the traffic flow data collected from the nearest stations on different time intervals and then the output combined with the incidents data is passed to RNN to predict flow on specific stations. They predicted the flow for a 30-min interval window by using the flow values of nearby stations for defined (e.g., four, five, etc.) time intervals. Data used for training and testing purposes are normalized using defined criteria. Five-minute interval vehicles flow data are obtained from PeMS [62] for training and testing of DL model. For validation purpose, root mean square error (RMSE) is used. Results show that they successfully predicted the flow values for specified stations on highways without using the historic data and were able to predict the flow in real time, but the method needs improvement as the predicted values are affected by different factors such as rush hours, holidays, any incident, etc. Thus, although the authors used the incident data, improvements are needed to make more accurate predictions. Data used for deep models are real, but the amount of data used for training the model is small because they used two months of five-min interval data for 60 consecutive locations for training and testing of deep model.

Ma et al. [30] used LSTM neural network to predict traffic speed for short time intervals. They used LSTM to predict speed on road networks for different short intervals of one to four minutes using historical data. Two-minute interval data from two sensors capturing traffic data on two opposite directions on a road are collected for one month (June 2013). MAPE and MSE are used as performance metrics and each model is executed 10 times to observe prediction consistency. Performance is compared with other models including Elman NN, time-delay NN, nonlinear autoregressive with exogenous input NN, SVM, ARIMA, etc. Two different type of input datasets are used for training, one of them includes historic speed values to be used as input for training and the other one includes historic speed and volume values as input for training. Results shows that the dataset using both speed and volume produces slightly better results than the results where only speed value is used. Furthermore, results show that LSTM performs much better than the other models and its results remains stable for all the time slots used for prediction purpose, but the other models improves their accuracy when time lags are increased. Results show a very high accuracy rate due to very low MAPE values but these could be high (MAPE) when time intervals are big. In addition, data are collected from only two sensors for a very short period thus they are almost uniform without any big change in the data and this could also be the reason for high accuracy rate.

Fu et al. also used LSTM and gated recurrent units (GRU) to predict vehicles flow using data from PeMS in [63]. They used one month of five-minute interval flow data collected from 50 sensors. The first three weeks of data are used for training and the last week of data are used for testing. Five-minute intervals data (flow values) for 30 min are used to predict the flow for next five minutes and the missing values in the dataset are replaced by historic average values. The prediction results are compared with the ARIMA and results are analyzed using MSE and MAE.

In another work, Jia et al. used a deep learning approach called deep belief networks (DBN) to predict the vehicles speed on a road network [25]. In this work, they used Restricted Boltzmann Machines (RBMs) for unsupervised learning and then used the labeled data for fine tuning. The dataset used is obtained from Beijing Traffic Management Bureau (BTMB). Three months of data (June–August 2013) are used which provide two-minute interval data collected from the detectors installed on a specified segment of road in Beijing, China. Around 11 weeks of data are used for training purpose, whereas the last week of data are used for testing purpose. This provides two-minute interval speed, flow and occupancy values and by using this two-minute interval data, the authors predicted speed for intervals of 2, 10, and 30 min. Furthermore, for performance analysis, three performance metrics are used: mean absolute percentage error (MAPE), root mean squared error (RMSE), and normalized root mean squared error (RMSN). No mechanism was mentioned by the authors to deal with the erroneous or missing data values and also no big data technology is used for data management. In addition, no specific information about data, e.g., number of detectors, etc., is given to know about the size of data. For deep model configurations, they executed the model with different configurations and, based on the MAPE values, they selected the best configurations. With best selected configurations, MAPE value for 2-min interval is 5.81, 7.33 for 10-min interval, and its value is 8.48 for 30-minute interval. This shows that it performs better for short time intervals and cannot cope with the stochastic fluctuations in long time intervals. Although results are quite good for speed prediction, how it behaves when some other information are included still needs to be investigated, e.g., we do not know whether data from multiple detectors are used or separate data for each detector are used because, in the former case, there would be more fluctuations in the data compared to the latter case. In addition, the size of data could also change the results.

A hybrid deep learning approach to predict traffic flow is proposed in [64]. In this approach, the authors combined convolutional neural networks (CNNs) and long short-term memory (LSTM) to use the correlation between the spatial and temporal features to achieve the goal of forecasting traffic flow. Data are collected from PeMS [62] that include 33 locations on one side of a highway. Fifteen months of five-minute interval data ew used. For comparison purpose, MAE are MAPE are calculated. In addition, to analyze the forecasting correctness of spatial distribution, average correlation error (ACE)is calculated. In this work, although they combined temporal and spatial features to get high accuracy, which they achieved, the method cannot be considered very effective as compared to the other approaches used in this work for comparison purpose. Use of both spatial and temporal features helps in improving the accuracy but they still need to investigate the best combinations to improve the accuracy.

Deep learning is also used for crash predictions on the road network. In [65], the authors proposed a dynamic Bayesian network approach to predict the crashes on highways in real-time by using the traffic speed conditions data. In this work, the relation between the crash incidents and the traffic states is established, so that it could be used to predict the possibility of crashes on highways. Traffic states on the crash site are divided into the upstream and downstream states. Here, upstream is the state just before the crash and downstream is the state just after the crash in traffic flow direction. A vehicle speed threshold of 45 km/h is defined to identify the free flow (FF), i.e., traffic state is FF if the vehicles average speed is above 45 km/h. Average speed below 20 km/h identifies the jam flow (JF) and it is considered congested traffic (CT) if the flow is between the FF and JF threshold values. By using these three states values, nine combinations (upstream and downstream) are defined to identify the occurrence of crashes in those states combinations. Crash reports data used in this work include 551 records where 411 records are used for training and the remaining 140 records are used for the testing purpose. A confusion matrix is created to see the results. Several metrics based on the confusion matrix data are used to analyze the prediction results. Best DBN accuracy reported in this work is 76.4% where the false alarm rate reported in this case is 23.7%.

Deep learning approaches are also used by many other researchers, who used them for traffic flow prediction [66,67,68,69,70,71,72,73]. Some others used it for traffic condition prediction on the road networks [29]. In addition to the vehicles flow data to predict traffic flow on highways, some researchers used other datasets with the flow dataset to predict vehicle flow on the roads. In [74], weather information is included with the input dataset, i.e., weather data from [75] are combined with the vehicles flow data to predict vehicles flow. In addition to the deep learning approaches for prediction, some researchers worked on traffic flow or travel time prediction using other approaches [76,77,78]. Therefore, we can say that this area has gained the focus of the research community, various approaches to predict the traffic behavior on road networks are used for effective traffic management. Among those approaches, prediction of traffic behavior by using deep learning approaches has a significant importance because of its ability to learn from many data and to learn the patterns more accurately as compared to the other approaches. By using deep learning, traffic behavior can be predicted by gaining high prediction accuracy but still it can be questioned as only a few methods use big traffic data as an input to the deep models. Deep learning with big input datasets requires more resources because of its highly compute intensive nature and also takes a lot of time for training. Thus, researchers try not to use big datasets for their deep models, thus not only compromising their accuracy but also restricting them to predict only for a specific duration by using the small input datasets. Because the training process takes a lot of time, no one has proposed a deep learning approach to predict the traffic behavior in real-time. To address the input dataset issue, we used a very large input dataset for more than 11 years so that our deep model could learn all possible trends of traffic data including a number of social and cultural events during this duration. In addition, to predict the traffic behavior in near real-time fashion, we used the pre-trained models to predict the behavior using the recent traffic data.

## 3. Methodology

In this section, we describe our prediction workflow and give a brief description of deep neural network that we used for prediction purpose in this work.

### 3.1. Prediction Workflow

**Figure 1:**
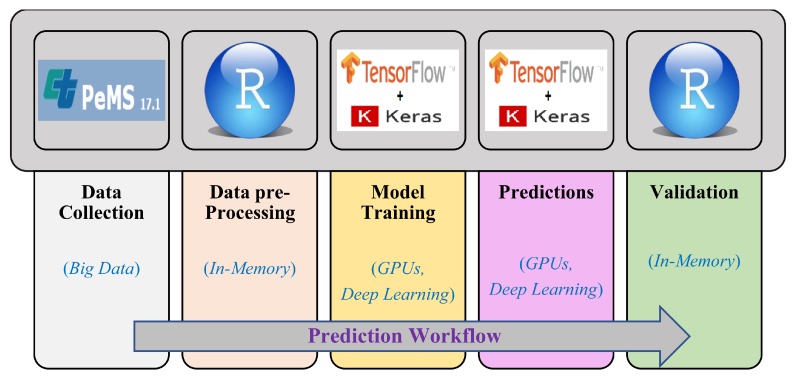
Prediction workflow.

In this section, we define our prediction workflow and the tools that we used in different phases to predict and analyze our prediction results. For traffic behavior prediction, we first need to get traffic data from a reliable source because the correctness of prediction results depends mainly on the data we feed as input to our deep model for training purpose. After collecting data from the data source in raw format, we have to analyze data to identify the attributes that could help us in prediction. This includes data cleaning and dealing with data veracity issues. In this phase, we have to search for the erroneous or missing data and have to replace those values with the correct or estimated values using different data techniques because it may affect the prediction results. After data cleaning/parsing phase, we have to define our deep model and have to feed the data to it for training process. After successful completion of training process, we can now get prediction results/output and compare these predicted values/output with the original traffic data to analyze the results and to calculate the accuracy of our deep learning model. This workflow with the tools used at different stages is shown in Figure 1.

### 3.2. Dataset

In this section, we discuss the dataset we used as an input to our deep learning models for traffic modeling. Table 1 gives the schema of the dataset used in our prediction model. These data provide enough information for different prediction and analysis purposes, including aggregate flow, occupancy, average speed and other values for all the lanes collected by the vehicle detector stations (VDSs). In addition to aggregate values, it provides lane wise data as well, as is clear from Attributes 7–38. Separate data for each lane are available for up to eight lanes. We used the dataset for corridor 01A: Los Angeles I-5. The length of this corridor is 13.784 miles and two directions on this corridor are denoted by I5-N and I5-S. For I5-N, there are 26 vehicle detection stations (VDSs) to collect data about the vehicles traveling on this route. In the opposite direction, i.e., I5-S, there are 25 such stations for data collection. Data are collected on five-minute interval basis and flow, occupancy, speed, and percent observed values are given for each five-minute interval. In this work, we only used data collected in one direction, i.e., I5-N.

**Figure 2:**
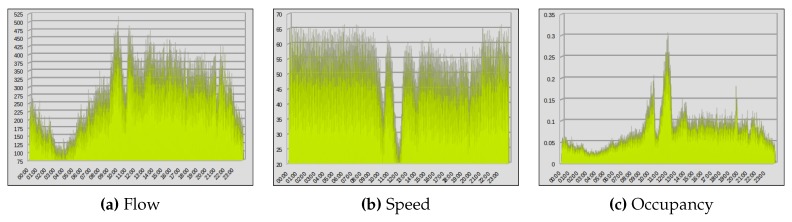
A depiction of vehicles data collected from PeMS (24 h) (November 2017).

These data provide enough information that could be used for different prediction and analysis purposes. In this work, we were interested in prediction of vehicles flow, speed, and occupancy at some specific VDSs and at specific time intervals. Therefore, we used only the data that provide us information about time, date, day, station id, vehicles flow, speed and occupancy values. In this work, we used the aggregate values that represent the overall value (e.g., flow) calculated by a VDS on the selected highway patch. In Figure 2 we show data containing one day of vehicle flow, speed and occupancy values provided by PeMS in November 2017. These data were collected from one selected VDS on the selected patch of the freeway I-5N.

### 3.3. Input Data Processing

As described in the previous section, our input dataset contains many parameters. Thus, the challenge here was to carefully analyze the dataset and select some of them based on the requirements of our deep learning models. Therefore, before using these data as input to our deep learning model, we had to extract the required information from the data and then to process some or all of its attributes to extract useful information. This process is called data parsing. In this process, we processed the dataset attributes values to extract some other useful information and made new attributes that were used as input to our deep learning model. In this work, we used some of the attributes values without making any change in their values, e.g., “StationId”, and processed some of them, e.g., “timestamp”, to extract some other useful information. We used “timestamp” attribute to extract hours, minutes, days, months, year, and day of week values. Extraction of useful attributes is a complicated process and it requires in-depth understanding of the input dataset attributes and their effect on the training process. In this case, we not only studied and analyzed the effect of each attribute in the dataset to make a balanced dataset, but also executed our deep model with different configuration and with different lists of input parameters values. Then by analyzing the results, we identified the attributes that were not useful for our deep model and also realized that we needed some other attributes to improve the accuracy. With the help of this long process, we finalized the input parameters for our input dataset. This data processing was done using *R* [79] and RStudio [80] and is given below in Algorithm 1. In this algorithm, we give all the steps to parse the dataset downloaded from the PeMS, so that it could be used by our deep model. We obtained data from PeMS in the text format and, after converting those data into csv format, we combined the data from all files (in this format, we merged data from 139 files) to one file for further processing. These are five-minute interval data that include many attribute values including “timestamp”. From “timestamp” attribute, we extracted different other attribute values, e.g., time, day, month, etc., and added these new attributes (columns) to the dataset. After this, we converted the data from long format to wide format based on, for example, flow values. To address the data veracity issue, all NAs in the dataset 3454 replaced with the values obtained using a predefined criterion. Finally, the data were normalized and divided into the training, testing, and prediction subsets to be fed to our deep neural network model.
**Algorithm 1** Input dataset parsing.**Input:** Multiple data files from PeMS archived datasets.**Output:** Parsed sub-datasets files to be used as training, testing, and prediction datasets.  1:  n←numberofinputdatafiles  2:  **for**
i=1,i++,i≤n
**do**  3:  $x_{i}\leftarrow loadData\left(file=i\right)$  4:  $rawData\leftarrow mergeDataFiles(x_{i})$  5:  **end for**  6:  X←ExtractRequiredInputParameters(rawData)  7:  **for**
j=1,toend_of_dataset
**do**  8:  $hours_{j},min_{j}\leftarrow extractTime\left(timestamp_{j}\right)$  9:  $date_{j}\leftarrow extractDate\left(timestamp_{j}\right)$10:  $date_{j}\leftarrow processDateVals\left(date_{j}\right)$11:  $day_{j},month_{j},year_{j}\leftarrow extractDMY\left(date_{j}\right)$12:  **end for**13:  ConvertDataFromLongToWideFormat()14:  weekDays←GetWeekDaysFromDate(date)15:  **for**
c=1,c++,c≤colsCount
**do**16:  **for**
r=1,r++,r≤rowsCount
**do**17:    **if**
$val_{cr}$ is NA **then**18:     $\begin{array}{c} val_{cr}\leftarrow average\left(val_{c(r-2)},val_{c(r-1)},val_{c(r+1)},val_{c(r+2)}\right) \end{array}$19:    **end if**20:  **end for**21:  **end for**22:  **for**
c=1,c++,c≤colsCount
**do**23:  colMax←getColMaxValue(c)24:  **for**
r=1,r++,r≤rowsCount
**do**25:    $val_{cr}\leftarrow val_{cr}÷colMax$26:  **end for**27:  **end for**28:  SplitDataIntoTrainingTestingPredSubsets()29:  writeDataOnFiles()

We used the five-minute interval flow value data. In one hour, we are 12 five-minute interval flow values and we used the first 11 flow values to predict the 12th interval flow values. The dataset we used for this model includes 139 months of data from January 2006 to July 2017. The schema of our input dataset is given in Table 2.

More than 42 million five-minute interval flow values were been used in our deep model. We divided the whole dataset into three subsets, training, testing, and prediction. We used the 8:1:1 ratio for training, testing, and prediction sub-datasets, i.e., 80% of data were used for training, 10% for testing, and the remaining 10% for prediction purpose. The ratio of the training and testing data split is discussed in the literature (see, e.g., [81]). With few data, finding an appropriate split ratio could be complicated due to the trade-off between the variance for parameter estimates (training data) and performance statistics (testing data). With many data, as in our case, the split was expected to make little difference. Ideally, it would be interesting to investigate the split practically using variations in the ratio. However, it will significantly increase the training and prediction computational time in our case due to the large amount of data. Nevertheless, we plan to investigate this issue in the future.

### 3.4. Deep Model Architecture

**Figure 3:**
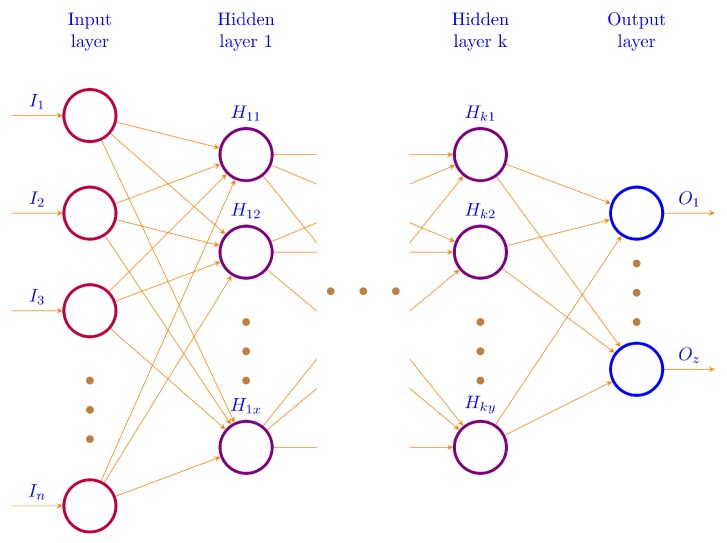
Architecture of a deep neural network model with one input, one output and *k* hidden layers.

In a neural network, many neurons are used in such a way that the output of a neuron could be used as an input to the other neurons in the network, as shown in Figure 3. Here, the left most layer is the input layer and the right most layer is the output layer. Number of neurons in input layer is the number of input parameters in our input dataset; since we were predicting traffic flow for a specific time interval, the output layer returned one single neuron considered as an output or predicted value. In all deep models used in traffic modeling section, *n* is the number of input parameters defined in respective sections in this paper. On the other hand, there was only one output parameter in all sections. The number of hidden layers is also mentioned in each section that defines the value of *k*. Similarly, the number of hidden units are also given with the other details. In this work, we used Keras [82], which avails the functionality provided by TensorFlow [83] at back-end for the execution of our deep learning model.

We give an overview of the execution of our deep model in Algorithm 2. We ran our deep model with different batch sizes and with different number of epochs. Therefore, instead of running our deep model with only one batch size and a fixed number of iterations, we developed a setup to run it multiple times with different combinations of configuration values. The purpose was to find the batch size and the number of epochs that gave us the highest accuracy. This way, we selected the bet suitable configuration values (e.g., batch sizes and number of epochs) for our deep learning model. In addition, we made different input dataset combinations with slight input parameters changes and ran them using all possible configuration combinations. This way, we could identify the parameters that influence the accuracy and thus we could change our input dataset accordingly. However, this was done at initial stage only, because once we finalized our input dataset, we used only that dataset and set the nDataType value to 1 as shown in the algorithm.
**Algorithm 2** Deep model algorithm.**Input:** Parsed input data for training.**Output:** Prediction results.  1:  nRepeatModel←10 {e.g., Repeat each model 10 times}  2:  nDataTypes←1 {If using multiple datasets}  3:  nDataCount←1  4:  batchArray←[100,200,500] {Different batch sizes}  5:  epochsArray←[100,200] {Set number of epochs}  6:  **while**
nDataCount≤nDataTypes
**do**  7:  $\begin{array}{c} trainX,trainY,testX,testY,predX,predY\leftarrow loadInputData() \end{array}$  8:  count←1  9:  **for all**nEpoch in epochsArray
**do**10:    **for all**nBatch in batchArray
**do**11:      **while**
count≤nRepeatModel
**do**12:      defineDeepModel()13:      compileDeepModel()14:      fitDeepModel()15:      evaluateDeepModel()16:      saveDeepModel()17:      makePredictions()18:      savePredictedValues()19:      saveAccuracyLoss()20:      count←count+121:      **end while**22:      count←1 {Reset repeat counter}23:    **end for**24:  **end for**25:  nDataCount←nDataCount+126:  **end while**

### 3.5. Validation

For performance evaluation of our deep models, where we were predicting the vehicles flow, speed, occupancy, number of passengers, etc., we used mean absolute error (MAE), mean absolute percentage error (MAPE), and root mean squared error (RMSE) to analyze the prediction results.

MAE is used to show the closeness between the actual and the predicted values, whereas MAPE shows the relative difference between the actual and the predicted values. MAPE is not suitable to calculate error rate if the input data or actual values contain zeros because in this case it suffers from the division by zero error. RMSE is used to calculate the standard deviation of the prediction errors. It tells how much scattered are the residuals and thus tells how far are the predicted data points from the regression line.

MAE, MAPE, and RMSE values are calculated by using the formulas given in Equations (1)–(3), respectively.

(1) $$MAE=\frac{1}{N}\sum_{i=1}^{N}\left|V_{i}-P_{i}\right|$$

(2) $$MAPE=\frac{1}{N}\sum_{i=1}^{N}\frac{|V_{i}-P_{i}|}{V_{i}}$$

(3) $$RMSE=\sqrt{\frac{\sum_{i=1}^{N}{\left(V_{i}-P_{i}\right)}^{2}}{N}}$$

In these equations, *N* is the size (number of values predicted by the model) of dataset used for prediction purpose, *V* is the set of actual values used as labels, and *P* is the set of values predicted by our deep learning model.

## 4. Experiments and Analysis

We used deep neural network to predict the traffic flow on a highway by using traffic data from PeMS, as described in the previous section. For prediction, we used different datasets and different model configurations. In addition, we also used our saved deep models to predict values on new datasets. For example, we used the saved pre-trained model to predict the vehicles flow and speed in the month of November 2017 that was not part of the input dataset used for training and testing purpose. This was helpful because training a deep model is a time consuming task. Thus, we could use the saved model to predict values for latest data in near real-time fashion. In the following sections, we discuss the details about each type of prediction model.

### 4.1. Traffic Flow Prediction

The specifications of the deep model used for prediction purpose are given in Table 3. As shown in this table, we ran the deep model with 17 input parameters, as defined in Table 2. There are four hidden layers in our deep model and the number of neurons in each hidden layer is also given in the configurations details. We executed this model with different batch sizes and using different numbers of epochs for each batch size, but here we only present the selected results with the configurations, as defined in Table 3. For analysis of results, performance metrics defined in Section 3.5 were used.

**Figure 4:**
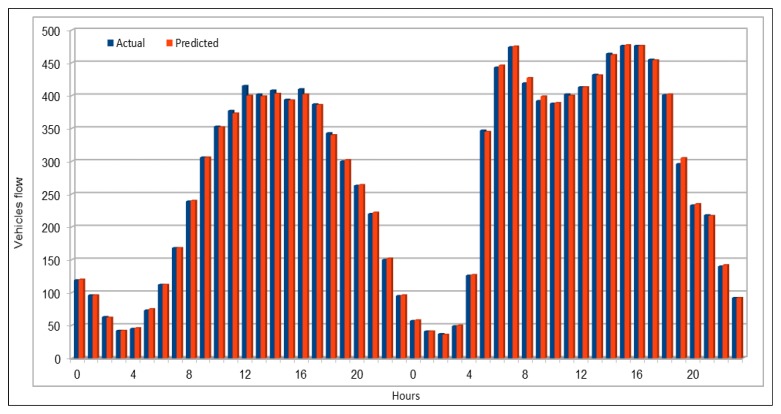
Traffic flow: Actual and predicted values for a single VDS.

**Figure 5:**
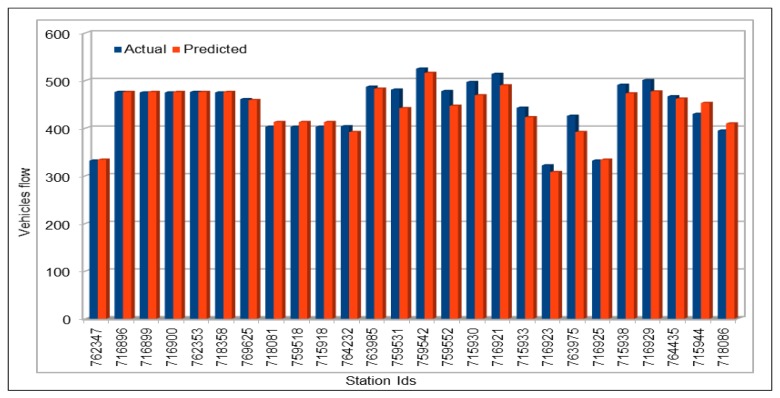
Traffic flow: Sctual and predicted values for all 26 VDS.

Figure 4 compares the actual vehicles flow values with the values predicted by our model. We used 10% of the dataset for prediction purposes (80/10/10 dataset split). Our prediction subset is very large and it is not possible for us to show the comparison between actual and predicted values for the whole set of predicted values. Therefore, we selected two days vehicles flow values for a specific VDS (VDS id 718086), on 30 and 31 July 2017. We can see that the predicted values are almost same as the actual values and both actual and predicted values bars look exactly the same. To compare of all the stations flow values with the predicted values, we compared the values of all the 26 VDS values for one hour flow on 26 June 2017, 16:00–17:00, as shown in Figure 5. The flow values were deliberately chosen for a peak hour.

**Figure 6:**
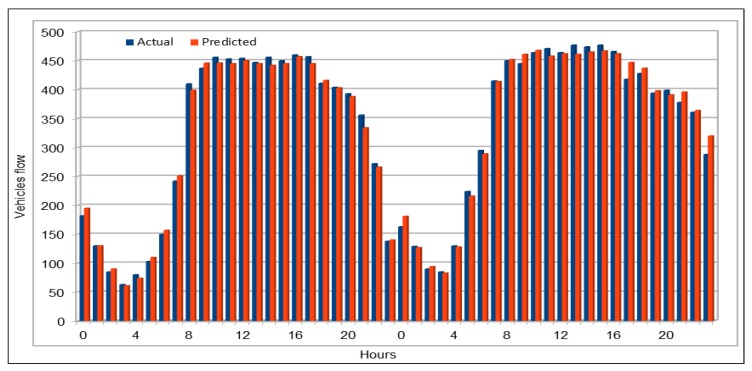
Traffic flow: Actual and predicted values (average of all VDS) (48 h).

**Figure 7:**
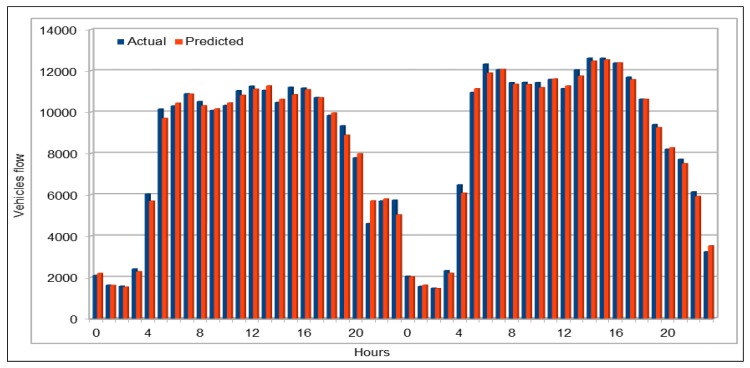
Traffic flow: Actual and predicted values (total of all VDS) (48 h).

Similarly, we also calculated vehicles average flow values for all VDSs for a duration of two days on 29 and 30 April 2017, as shown in Figure 6. For this, we calculated the average flow values of all the VDS for each of 48 h for two days. In addition, two days of vehicle total flow values for all VDS for a duration of two days on 30 and 31 May 2017 are shown in Figure 7. The purpose of comparing these values using one hour data, average and total data for all VDS for more than one day and selecting different dates and times for collecting data was to give readers an overview of our prediction results in different ways. The results from randomly selected data for all VDS show that the our deep model completely followed the trend of original values and accurately predicted vehicles flow values.

**Figure 8:**
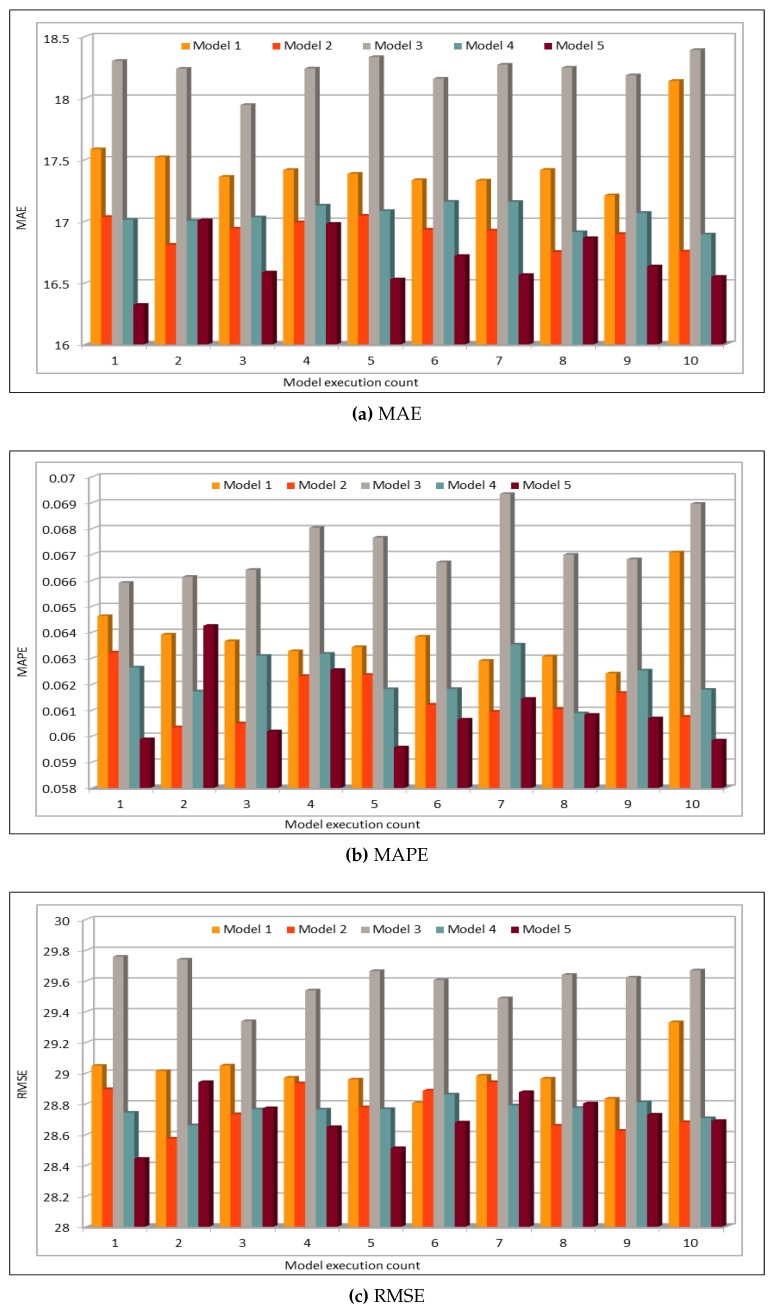
Traffic Flow: MAE, MAPE, AND RMSE Evaluation Metrics.

In addition to comparing the actual and predicted flow values, we also used well-known evaluation metrics to calculate the prediction credibility. In this work, our deep learning model model was executed 10 times. Figure 8 shows the variation in the prediction error rates calculated using the defined performance metrics. This figure compares the results of five deep learning models with different batch sizes and numbers of epochs, as shown in Table 3. Model 1 in this figure represents the results of model with batch size of 500 and number of epochs is 100. Similarly, Model 5 represents the results of deep learning model with batch size of 5000 and number of epochs is 1000. We obtained the minimum MAE value 16.32, whereas the maximum value is 17.00. As MAE calculates the absolute difference between the actual and the predicted values, we can say that MAE values show very good results because, in our dataset, vehicle flow values are very big, i.e., in hundreds or thousands. Normally, these are more than 500. Thus, for values that are normally bigger than 500, a difference of 16 or 17 is negligible and, because the flow values are even higher, it proves that the accuracy rate is very high and error rate is very low.

### 4.2. Traffic Speed Prediction

We discuss the results for the speed prediction in this section. We used the same data collected from PeMS [62], as defined in Section 3.2. The schema of the speed dataset is the same as detailed for the traffic flow data (see Table 2). Parameters 1–6 are same for both the flow and speed datasets. Parameters 7–18 are different in that these represent the five-minute interval vehicle speed values. PeMS defines speed as the average speed of vehicles passing through a vehicle detection station (VDS). The entry speed_00 defines the average vehicle speed calculated at a VDS during the first five minutes of an hour. The entry speed_55 defines the average speed value at a VDS during the last five minutes of the hour. There are a total of ten entries in between for each hour. We used the same performance metrics for speed as defined in Section 3.5.

**Figure 9:**
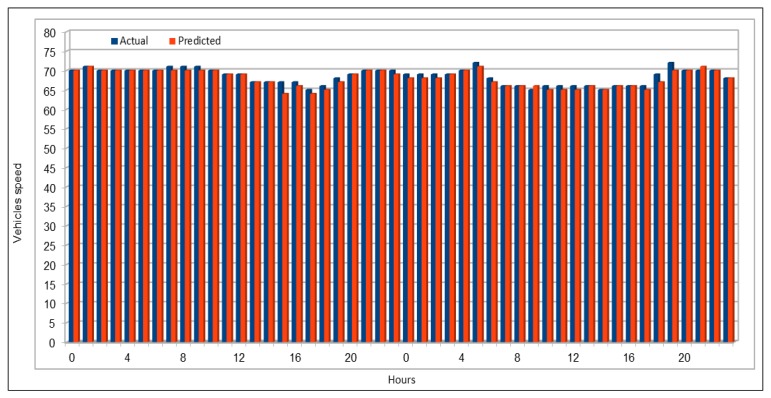
Traffic speed: Actual and predicted values for a single VDS.

**Figure 10:**
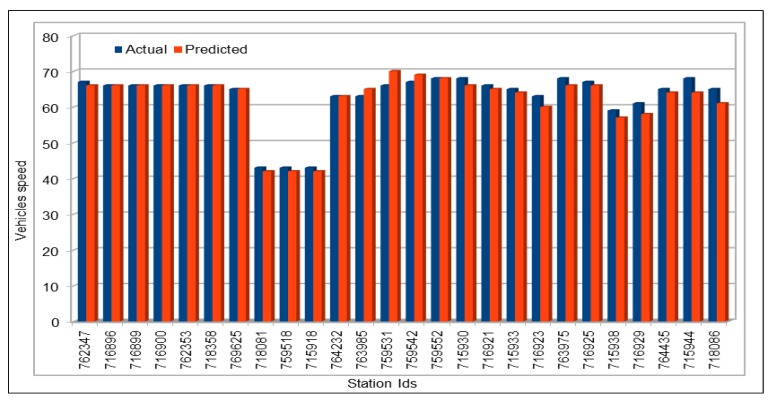
Traffic speed: Actual and predicted values (all VDS) (peak hour: 26 June 2017, 16:00–17:00).

**Figure 11:**
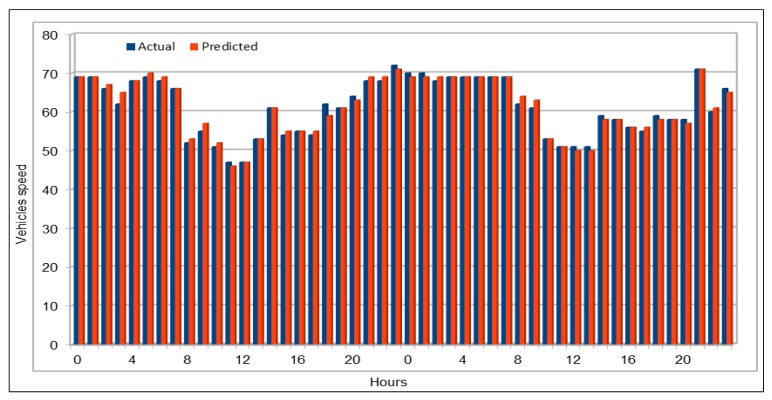
Traffic speed: Actual and predicted values (average of all VDS) (29 and 30 April 2017).

Figure 9 shows the comparison of actual and predicted vehicles speed values. Data for comparison were collected in the same way as we did before for comparison of vehicles flow. We compared 48 speed values and the highest difference between the actual and the predicted values was 3, which only occurred once, while values with a difference of 2 repeated only two times, 18 values show a difference of 1 and the remaining 27 values are exactly same. In most cases, the actual and predicted values are exactly the same. In the cases where the predicted values are different from the actual values, these differed by one at most, and this difference is inconsiderable given that the speed in these cases was above 60. Figure 10 compares the actual and predicted speed values on 26 June 2017, 16:00–17:00, for the 26 VDS. The flow values were deliberately chosen for a peak hour. The plots show near equal similarities between the actual and predicted values. Figure 11 plots the actual and predicted speed values for two days, 29 and 30 April 2017. These are the average of all the respective speed values of the 26 VDS. Note in the figure that, in all cases, the predicted values of the vehicle speeds are very similar to the actual values and the difference are negligible.

**Figure 12:**
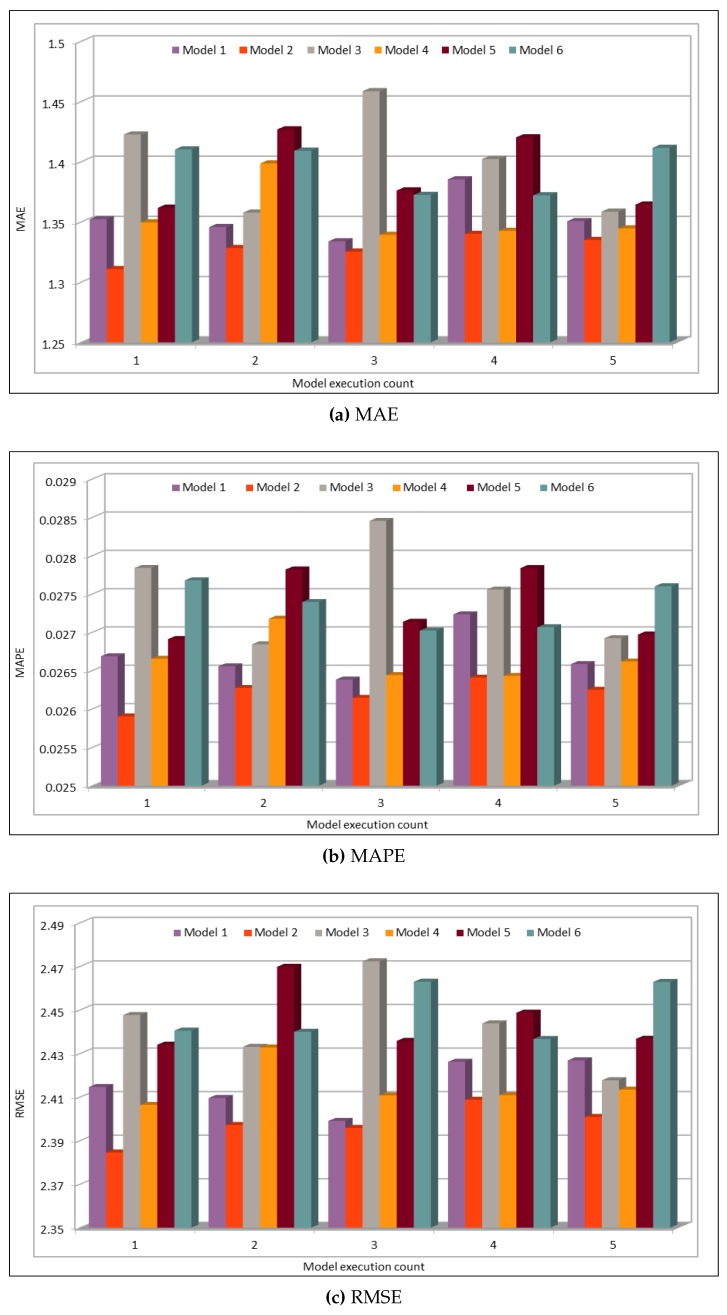
Traffic speed: MAE, MAPE, and RMSE evaluation metrics.

The model evaluation results are shown in Figure 12. We plotted the results of six different model configurations with different batch sizes and numbers of epochs. The models were executed five times to ensure the consistency of the prediction results. The deep model configurations for these and all the speed predictions in this subsection are given in Table 4. The MAE, MAPE, and RMSE results in the figure indicate an excellent similarity between the actual and predicted speed values. We executed the model five times to see the variations in prediction results, thus we could get the minimum, maximum, and average MAE error values. The minimum MAE value obtained is 1.31, whereas the maximum MAE value is 1.46. Normally, the average speed values are above 50, thus a deviation of 1.3–1.45 in these values does not make a difference and the results could be used to reflect the average speed on the freeway. Similarly, MAPE values are also very low where the minimum MAPE value is 0.0258 and the maximum value is 0.028. Therefore, MAPE values show a deviation of 2.58–2.8% from the actual speed values. This performance metric also shows very low error rate and it could be neglected. Similarly, RMSE error values range from 2.38 to 2.47 and thus confirm that the deviation rate is very low and the prediction results shows the same trend as shown by actual speed values.

### 4.3. Traffic Occupancy Prediction

This section presents an analysis of the prediction results for traffic occupancy. For this purpose, we used the same vehicles dataset (see Table 1) as we did for experiments reported in Section 4.1 and Section 4.2. The schema of the input dataset used for the occupancy prediction is also the same as used for traffic flow and defined in Table 2. Parameters 1–6 are the same in both the flow and occupancy cases. Parameters 7–18 differ in the same way as before in the case of speed, i.e., they represent the five-minute interval of vehicles’ average occupancy values. PeMS [62] defines occupancy as the amount of time it takes for a vehicle to pass through a VDS deployed on the freeways. The entry occupancy_00 represents the average vehicle occupancy calculated at a VDS during the first five minutes of an hour. The entry occupancy_55 represents the average occupancy value at a VDS during the last five minutes of the hour. There are ten entries in between for each hour. The evaluation metrics used in this section are the same as defined in Section 3.5.

**Figure 13:**
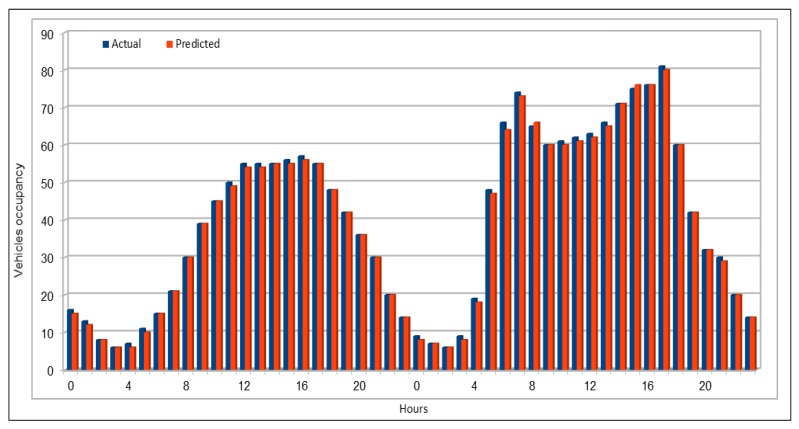
Traffic occupancy: Actual and predicted values for a single VDS.

**Figure 14:**
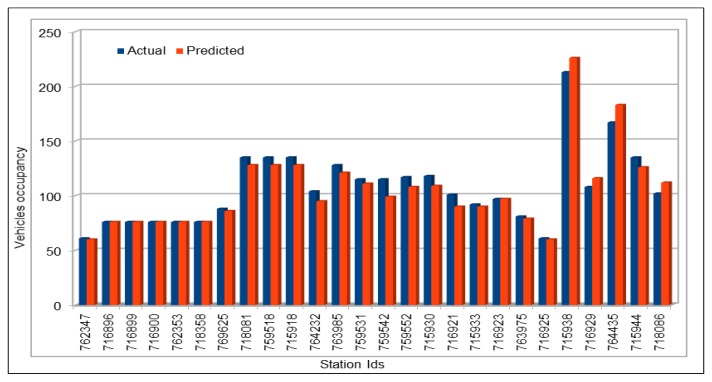
Traffic occupancy: Actual and predicted values (all 26 VDS) (peak hour).

**Figure 15:**
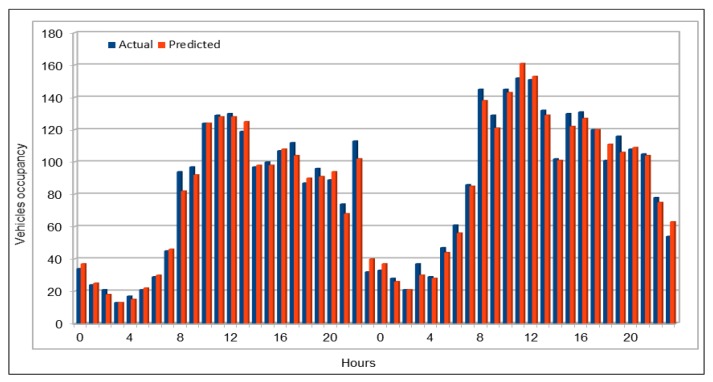
Traffic occupancy: Actual and predicted values (average of all VDS) (48 h).

Figure 13 compares the actual and predicted occupancy values. Because the occupancy values are very small, we multiplied both, actual and predicted values, by 1000. Comparison of actual and predicted values showed that our model presented the same trend in making predictions as the actual occupancy values. As we did before in vehicles flow and speed cases, we compared the occupancy values of all the 26 VDS values for one hour on 26 June 2017, at 16:00, as shown in Figure 14. In addition, vehicle average occupancy values for all VDSs for a duration of two days on 29 and 30 April 2017 are shown in Figure 15. Comparison of values in vehicles occupancy case also show that the predicted values are very close to the original values and these could be used to represent the original values if required.

**Figure 16:**
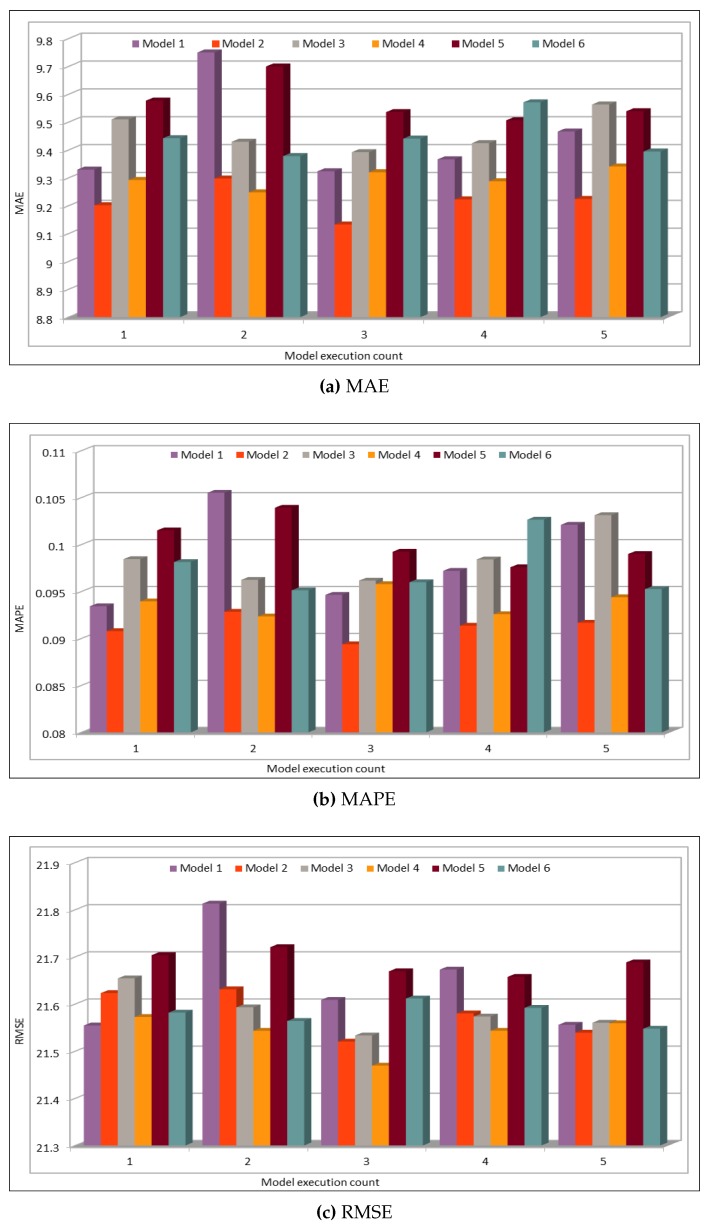
Traffic occupancy: MAE, MAPE, and RMSE evaluation metrics.

Figure 16 shows the MAE, MAPE, and RMSE values for the occupancy prediction. Similar to the analysis presented for the traffic flow and speed prediction in the previous subsections, six different deep learning models based on the batch sizes and epoch values were executed. We executed our deep learning model five times using the configuration settings defined in Table 5. Note the variations in the error rates while analyzing the predicted values using the three evaluation metrics. Note that the minimum MAPE value obtained by executing the model five times was 0.088, whereas the maximum value was 0.105. The error rates in vehicles occupancy prediction are relatively higher than those for the flow and speed values. This could be due to the very small occupancy values, which are in decimals because rounding off a very small value to a specific number of digits affects the prediction accuracy.

### 4.4. Comparative Analysis

In this section, we briefly compare our prediction results with some of other state-of-the-art works in this area. It is very important to compare the prediction accuracy or error rate, but this is not the only factor that could be used to show the effectiveness of an approach used for prediction. While working in this area, we found that the prediction results may differ even if different traffic data attributes were predicted. Thus, only comparing the result on the basis of statistical methods for performance comparisons is not enough. There are some other factors that not only affect the forecasting process but also provide the basis to choose the best approach even if they have same prediction accuracy. These are defined as follows.

1. **Prediction Method:** It gives an idea about different prediction techniques and we can identify which of them performs better under which circumstances.
2. **Prediction Attribute:** Which traffic data attribute has been predicted using the proposed method. Prediction accuracy may differ while predicting different traffic data attributes.
3. **Input Dataset:** What is the source of input dataset? This is important because it tells about the way it is collected and we can know about the data health.
4. **Dataset Size:** How big your input dataset is in terms of number of records in it. This is important because especially for deep learning it affects the training process.
5. **Data Collection Duration:** It makes the data more diverse because if we are collecting, e.g., one month data, then our deep network may not know about the events/factors that occurs in other months.
6. **Evaluation Criteria:** This is the metric used to compare the values obtained by using different performance metrics, e.g., MAE and MAPE.

Table 6 compares our prediction results with the other works. As mentioned above, we not only compared the values obtained by calculating error rates such as MAPE, RMSE, etc. but also compared our work with other works in light of other factors as defined above. Zhao et al. [26] used LSTM for to train the deep model with many data, 26 million records approximately. In this work, they also used MAPE for prediction error calculation. As compared to this work, we used more than 42 million records to train our deep neural network and our flow prediction results are much better than their results. They predicted the flow for each separately, which makes their data uniform and thus helps them to get low error rate, and their minimum reported MAPE value is 6.05 for 15-min interval. On the other hand, we used the data from 26 VDSs, and instead of calculating error for a single VDS, we calculated it for all. Even after including non-uniform data from different VDSs, our minimum MAPE value is 5.96 for 5-min interval values (Figure 8). In other cases, where we used pre-trained model for prediction, MAPE values are even less than 4 (see Figures 20 and 22). Lv et al. [23] also used quite big data, bu for different highways. Data for one highway can only be used to predict flow on that highway and they are not useful for others. Therefore, for each highway, their dataset is very small, in terms of both size and duration. In addition, MAPE values reported in this work are very high as compared to our MAPE values, as discussed above. Ma et al. [30] predicted vehicles speed. Their input dataset is very small in terms of both size and duration. They have reported minimum MAPE 3.78 (using 2-min interval data) while using speed for four different time intervals (1 min, 2 min, 3 min, and 4 min) data for one detector. MAPE values for other detectors are much higher than this value. However, in our case, we reported MAPE of 2.59, which is far less than their reported results. Results reported in [84] are also calculated using PeMS flow data but the authors calculated the mean accuracy using the formula MA = 1 − MAPE. Maximum accuracy shown in figures in this work is 0.91, with the minimum MAPE value of 9, which is very high as compared to our MAPE results. In addition to making comparison with the other models, we compared our results with LSTM and SVM models as well by using the same input data for those models. We used speed data for comparison purpose and only present y MAPE values to compare the results with our deep model. We can see that MAPE values calculated using our model are far less than the results when we executed LSTM and SVM models using the same input data. In LSTM case, we noticed one more thing: there was no consistency when we executed LSTM model multiple times. In some cases, we obtained good results and in some cases we obtained very bad results where the MAPE values were showing 100% error rate. On the other hand, our model not only showed high accuracy, but also our prediction results were consistent when we executed our model multiple times.

### 4.5. Prediction from Pre-Trained Deep Models

Training a deep model is a compute intensive and time taking job. In some cases, as we did for vehicles flow prediction (Section 4.1), it takes days to train a model. Thus, to make predictions in real-time or close to real-time fashion, we can use the pre-trained models for traffic behavior prediction. We used our pre-trained model to predict the traffic flow on selected highway during for some specific time, day, week or for some specific duration. For this purpose, we predicted vehicles flow on weekends (Saturday and Sunday), morning peak hours, and evening peak hours on a specific day. In the following subsections, we present the results obtained by predicting values using the pre-trained models.

#### 4.5.1. Traffic Flow Prediction: Weekends

**Figure 17:**
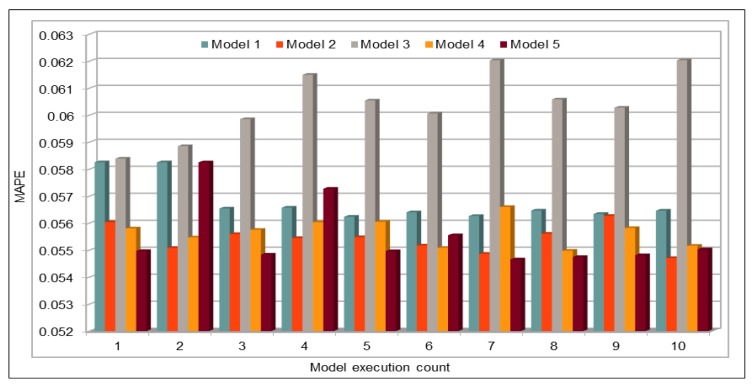
MAPE calculated by comparing actual and predicted vehicles flow on weekends.

**Figure 18:**
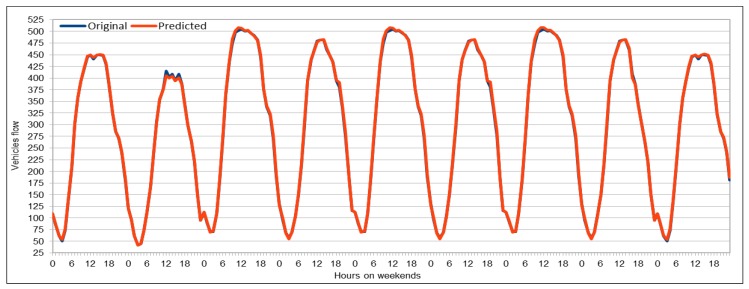
Comparison of original and predicted flow values on weekends.

We used weekends (Saturday and Sunday) data for three months, September–November 2017, to predict vehicles flow on the selected patch of the freeway I5-N in the region of Los Angeles County. We obtained the predicted values by running the same model (see Table 3), thus we used the same dataset schema (see Table 2). Again, as we did for flow and speed models, we executed the saved models multiple times (10 times in this case) to analyze the results and variations in predictions. Thus, we compared minimum and maximum error value calculated while evaluating the results. As shown in Figure 17, minimum MAPE error value is 0.0547 and the maximum MAPE error rate is 0.0583, which shows just 5% error rate. Note that we did not predict the vehicles for only one selected weekend but we predicted it for three months (September–November 2017). Thus, we can say that, if we predict the flow for only one weekend, we would get even better results because of less variation in the input data. Figure 18 compares the original vehicles flow and the predicted vehicles flow on the weekends for these three months. The blue plot in the figure represents the actual flow values, whereas the predicted values are represented by the orange plot. The traffic flow plots clearly show the typical diurnal pattern of road traffic on weekends where the flow repeats the lowest and peak values on similar times each day of the weekend. Comparing the two plots, we note that both actual and predicted value plots are so close that most of the two plots appear as one single graph. This is because the predicted values are either the same as the actual values or are very close to the original flow values. We compared these values on weekends and diurnal patterns of both actual and predicted values are identical to each other. It is clear that our model successfully predicted the vehicles flow on the weekends except on maximum flow values, where the predicted flow values are slightly smaller than the original flow values. However, this is negligible for two reasons: (1) there are only few cases where there are peak flow values are recorded; and (2) in most cases, the number of vehicles is more than 500, thus a predicted difference of 20–30 vehicles can be tolerated.

#### 4.5.2. Traffic Flow Prediction: Morning Peak Hours

**Figure 19:**
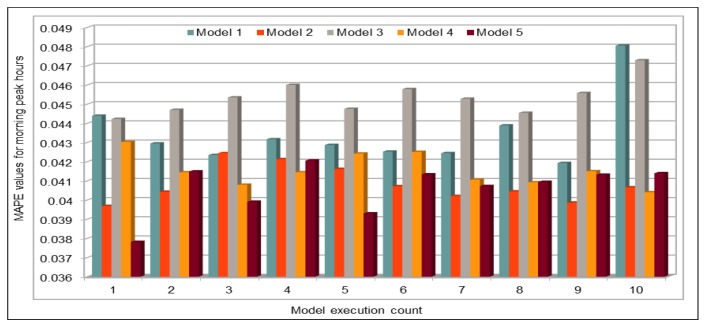
MAPE calculated by comparing actual and predicted vehicles flow on morning peak hours.

We collected the data for only few hours (from 06:00 to 10:00) on 30 November 2017 to predict flow values during morning peak hours. We used the same trained model to predict the flow values. The results presented here are quite interesting and we managed to get very low MAPE values for first time, as shown in Figure 19. The model with the same configuration was executed 12 times and we obtained very low MAPE values as compared to the previous executions of our deep models with other configurations and data. In this case, we can see that the minimum MAPE value obtained was 0.0378 for the first execution of this model and the maximum or highest MAPE value was 0.0423 when the model was executed for 11th time with the same configurations. If we compare the results of all 12 executions, on average the MAPE% value was 4.07%, which is a good achievement for the expressway where the range of flow values is 250–592. To analyze results, we also calculated the minimum and maximum flow values from the results obtained from the first execution of the model (out of 12 executions). The prediction results show that minimum flow value predicted by our model was 248 and the maximum value predicted by this model was 617. Therefore, we can conclude that the results obtained from the model are very close to the actual values collected from the sensors installed on VDSs and our predicted results could be used to predict the actual situation on those roads.

**Figure 20:**
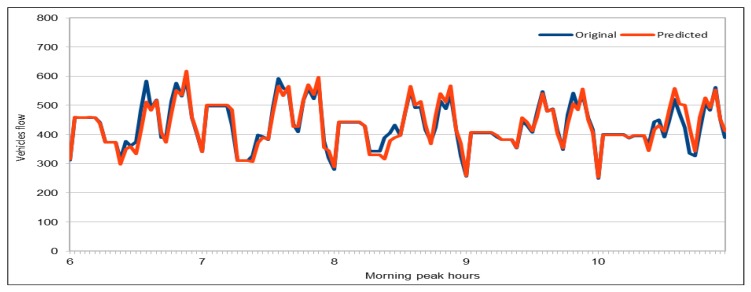
Comparison of original and predicted flow values on morning peak hours.

Figure 20 plots the original and predicted flow values. The predicted values are very close to the original values. These differ from the original values in some cases slightly but mostly the predicted values are very close to them. The blue curve in this figure represents the original vehicles flow values, whereas the orange color represents the vehicles flow values predicted by our pre-trained model. We can see that both curves are almost identical and both show the same trend. In few cases, there is a difference between the original and predicted values curve, and in only two cases the difference between the original and the predicted values is more than 50. Except these two cases, the whole curves are represent the same trend and show that, in most cases, the flow values were very close to the original values. Therefore, we can say that our predicted values accurately represent the actual flow condition on the freeway and can effectively be used while taking decisions for traffic management on the freeway. We believe that the prediction difference of approximately 70–80 against the actual value of 580–590 in the early morning (left side of the figure) is due to one of those anomalies and non-recurrent behaviors that are common in road traffic environments due to accidents and other uncertainties. These naturally become more critical to predict in rush hours, as in the case here. While the overall results of our deep learning model are promising, we do acknowledge that further investigation in needed to create more robust prediction models which give highly accurate predictions under non-recurrent situations and rush hours. We plan to look into this in the future.

#### 4.5.3. Traffic Flow Prediction: Evening Peak Hours

**Figure 21:**
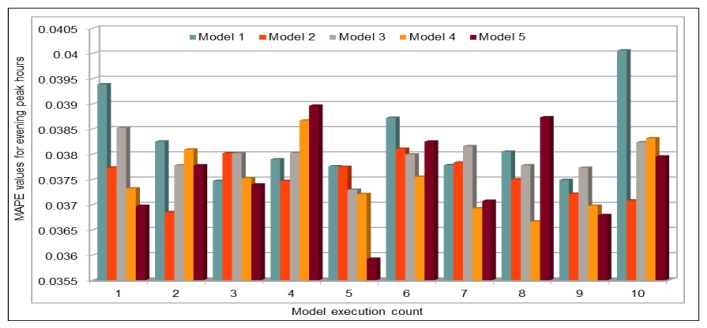
MAPE calculated by comparing actual and predicted vehicles flow on evening peak hours.

Again, by using the pre-trained model, we predicted the vehicles flow on a freeway during peak hours in the evening (14:00–18:00) on 30 November 2017. We obtained the dataset with the same attribute values as used in training process. As there are 26 VDSs on our selected freeway and this experiment used only five hours on one day (30 November 2017) of five-minute flow value data, i.e., 130 records in total were used for prediction. In Figure 21, we can see that we obtained very high accuracy because the MAPE values for all 12 executions of our deep model with the same configurations are not more than 0.039. Minimum MAPE value shown in this figure is 0.0359 that was obtained while executing the model for 5th time and the maximum value is 0.039 that was obtained while executing the model for 4th and 11th time. Therefore, the lowest MAPE% value is 3.59 and the highest MAPE% value is 3.9. For further analysis of predicted values, we calculated the minimum and maximum vehicles flow values for both real data and predicted flow values. Minimum and maximum vehicle flow values collected from the sensors on the 26 VDSs during these five hours were 264 and 612, respectively. Minimum vehicle flow value that was predicted by our pre-trained deep model was also 264, which is the same as in the original dataset. On the other hand, the maximum predicted value is 581, whereas the maximum value in the original data was 612. Although there is a difference of 31 vehicles, it could also be accepted because there were only two values in original data that were bigger than the maximum predicted vehicle flow value. In addition, it does not make a big difference because we can say that at maximum there was a difference of only 5% in one case, which is negligible when number of vehicles is more than 500.

**Figure 22:**
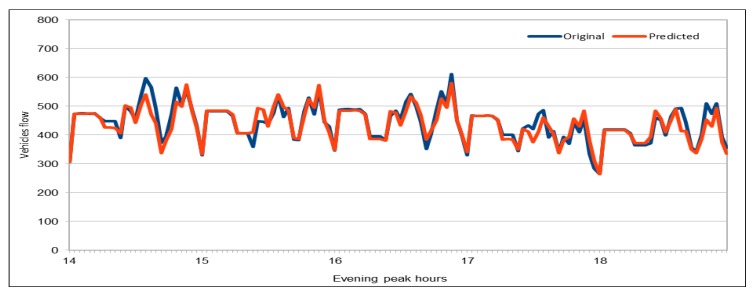
Comparison of original and predicted flow values on evening peak hours.

Figure 22 shows a comparison of the original and predicted flow values. As we showed for the morning peak hours case, the prediction results are very close to the original values. As observed in the comparison of actual and predicted values during the morning peak hours, the predicted values show the same vehicles flow trend during the evening peak hours as well. Although there is a difference between the actual and predicted flow values, both curves show the same flow trend in this case as well and hence these values could also be used for traffic management on the selected patch of the freeway I-5N.

## 5. Performance Enhancement for Real-Time Prediction

### 5.1. Real-Time Prediction Using Pre-Trained Deep Models

**Figure 23:**
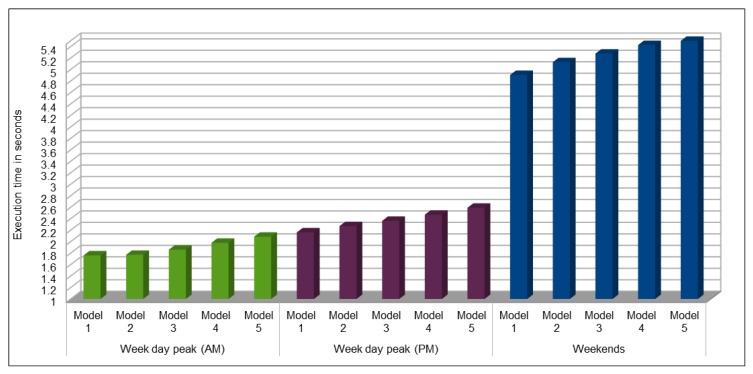
Model execution time while making predictions using the pre-trained models.

Traffic modeling is very useful in traffic management tasks where predicted traffic flow, speed, occupancy, etc. results can be used by the management authorities for traffic management in real-time. Traffic data are mostly available in archived form, i.e., historical traffic data, which is helpful for analysis purpose but is not useful in the case of disaster or any other emergency situation. To the best of our knowledge, no source provides real-time vehicle data. Thus, we could use our pre-trained models to predict the vehicles flow, speed, occupancy, etc. on highways in near-real time manner. In Section 4.5, we present the prediction results by using the pre-trained models during morning peak hours, evening peak hours, and on the weekends. As we were only making predictions using the pre-trained models, it was very quick and we obtained very quick prediction results. For prediction from pre-trained models, it directly depends upon the size input dataset to make prediction. If we want to make predictions for very short time span, it would be very quick and we could get results in microseconds. As show in Figure 23, while predicting vehicles flow during morning and evening peak hours, input dataset was small as compared to the input dataset while predicting the flow on a selected VDS on weekends in a month. Therefore, we were able to predict the flow values in less than 2.5 s while predicting it for a period of four hours, but it took around 5 s to predict the values for weekends. This shows that we can make predictions for each VDS in near real-time fashion on hourly or 30-min interval basis.

### 5.2. Comparison of Deep Model Execution Time on CPUs and GPUs

**Figure 24:**
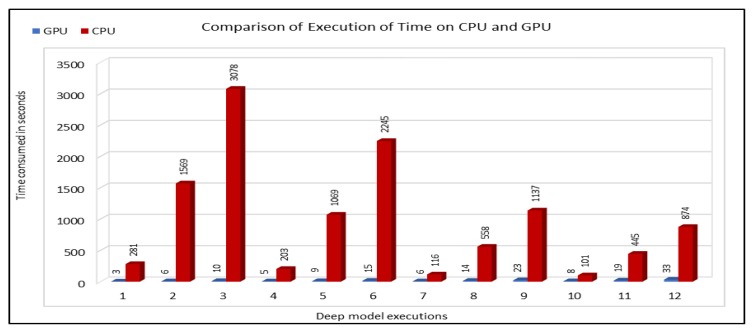
Comparison of deep model execution time on CPUs and GPUs.

Because of compute intensive nature of learning models, a lot of time is consumed in model training. Model training time increases with the increase in the size of the input dataset used for training purpose. Therefore, it affects the importance of the predicted values, especially in cases where training time continues for hours on normal computers. Thus, we used GPUs to reduce the model training time and to get the prediction results in an efficient manner. This also enabled us to make predictions in near real-time and thus our prediction results could effectively be used by traffic management authorities. We compared the execution time on CPU and GPU in seconds. For execution time calculation on CPU, we executed the model on a PC with Intel(R) Xeon(R), E5-2640 @ 2.50 GHz with a capacity of 4 GHz, with 16 GB RAM. For GPU, our models were executed on Quadro 6000 GPU from NVIDIA. Figure 24 compares the execution time on both these devices where the model execution time is given in seconds. Note that, in this comparison, we used three months of vehicles occupancy data for training the models. We compared the training time for 12 different model configurations while using those input data. We used small input dataset for time comparison, because this way we could easily elaborate the differences between the consumption times on both CPUs and GPUs.

From the execution time comparison graph, we can see that there is a huge difference between the execution time on CPUs and GPUs. It took only few seconds while training the models on GPUs, whereas CPUs took hundreds or thousands of seconds while training the same models. Even in the cases where CPU took less time for training, GPUs took less than 5% of the execution time, e.g., Models 7 and 10.

## 6. Conclusions and Future Work

Smart cities appear as “the next stage of urbanization, subsequent to the knowledge-based economy, digital economy, and intelligent economy” [1,32]. Road transportation is the backbone of modern economies, albeit it costs 1.25 million deaths annually. Trillions of dollars of the global economy are lost due to road congestion in addition to the congestion causing air pollution that damages public health and the environment. Road congestion is caused by multiple factors including bottlenecks on the road networks, road traffic crashes, bad weather, roadworks, poor traffic signal control, and other incidents. Better insights into the causes of road congestion, and its management, are of vital significance to avoid or minimize loss to public health, deaths and injuries, and other socio-economic losses and environmental damages.

Many road traffic modeling, analysis, and prediction methods have been developed to understand the causes of road traffic congestion, and to prevent and manage road congestion. The forecasting or prediction of road traffic characteristics, such as speed, flow and occupancy, allows planning new road networks, modifications to existing road networks, or developing new traffic control strategies. Deep learning is among the leading-edge methods used for transportation related predictions. However, the existing works are in their infancy, and fall short in multiple respects, including the use of datasets with limited sizes and scopes, and insufficient depth of the deep learning studies. Further work is needed on the use of deep learning for road traffic modeling and prediction. Moreover, the rapid ICT developments such as high performance computing (HPC) and big data demand for non-stop innovative. multidisciplinary, uses of the cutting-edge technologies in transportation.

This paper addresses the challenges of road traffic prediction by bringing four complementary cutting-edge technologies together: big data, deep learning, in-memory computing, and HPC. The approach presented in this paper provides a novel and comprehensive approach toward large-scale, faster, and real-time road traffic prediction. The road traffic characteristics that we have predicted are flow, speed, and occupancy. We used Convolutional Neural Networks (CNNs) in our deep learning models for the training of deep networks using over 11 years of PeMS road traffic data from 2006 to 2017. This is the largest dataset that has been used in a deep learning based study. Big data veracity issues have been discussed in detail and methods to address the incompleteness and errors in data have been described. Several combinations of the input attributes of the data along with various network configurations of the deep learning models were investigated for the training and prediction purposes. These multiple configurations show consistency of the accuracy of the results.

The use of GPUs provides a speedy deep learning training process, and this has been demonstrated by comparing the execution time performance of the training process on GPUs with CPUs. The possibility of real-time prediction by using pre-trained deep learning models have also been discussed in detail. The accuracy of our deep models was investigated using MAE, MAPE, and RMSE. The results demonstrate remarkably high accuracy. The comparison of actual and predicted values of the road traffic characteristics have also added to the insights.

The future work will focus on considering larger datasets; investigating different combinations of flow, occupancy, speed and other road traffic characteristics, in order to enhance prediction accuracy; improving prediction methodologies and analytics; using diverse types of road traffic datasets; fusing multiple datasets; and using multiple deep learning models.

We have explored the possibility of real-time prediction by saving the pre-trained deep learning models for traffic prediction using larger dataset, and subsequently using it on smaller datasets for near real-time traffic predictions using GPUs. This was a first step towards the real-time prediction of road traffic and it will be further explored in our future work with the aim to develop real-time predictions for dynamic road traffic management.

An important concern in predicting road traffic is the credibility of the prediction model under non-recurrent situations, particularly in those situations where additional external data—such as incident data—are not available or could not be incorporated in the prediction models. In this study, we used 11 years of traffic data over a fairly large stretch of freeway, which we believe incorporate the non-recurrent road traffic behavior in a considerable manner. For instance, the use of traffic data for a shorter period of time is very likely to lead to poor training in terms of recurrent behavior. Nonetheless, predicting non-recurrent behavior is a challenge and, even in our case, despite the use of data of very long duration, we have seen in some instances relatively higher error rates during rush hours (see Figure 20). It will be a challenge for the pre-trained models too and would require retraining of the deep learning models with every emerging non-recurrent traffic behavior. This topic needs further investigation in the future and we are very hopeful that the concerted efforts of artificial intelligence and other scientific communities will continue to improve methods for predicting non-recurrent behavior.

The study presented in this paper used 11 years of road traffic data for training and prediction purposes. The practice to collect road traffic inductive loop data has become a norm in several developed countries such as in Europe and the US. They are being collected now for over a decade in countries such as the UK and the US. However, many countries around the world, and particularly in urban environments (in contrast to freeways), inductive loop or other road traffic data are not available in such large quantities. Therefore, the conclusions presented in this paper cannot be generalized. Future work will look into this.

Finally, the paper contributes novel deep learning models, algorithms, implementation, analytics methodology, and software tool for smart cities, big data, HPC, and their convergence. The paper also serves as a preliminary investigation into the convergence of big data and higher performance computing [60] using the transportation case study. These convergence issues will be further explored in the future with the aim to devise novel multidisciplinary technologies for transportation and other sectors.

## Figures and Tables

**Table 1:** Schema of vehicles dataset.

No.	Attribute Name	Description
1	Timestamp	Defines the time when data is captured at a vehicle detector station (VDS). Timestamp gives both date and time when data was calculated at a specific VDS.
2	StationId	Id of a vehicle detector station (VDS). In these data, station id is a numeric value.
3	StationTotalFlow	Total number of vehicles passed through a specific VDS at a specific time interval.
4	StationAvgOcc	Average occupancy rate; calculated at a VDS at a given time interval defined in timestamp attribute.
5	StationAvgSpeed	Average speed calculated at a specific VDS at specific time interval.
6	StationPercent Observed	Number of lanes at this VDS station
7	Lane1TotalFlow	Number of vehicles in Lane1
8	Lane1AvgOcc	Average occupancy calculated w.r.t. lane1
9	Lane1AvgSpeed	Average speed calculated at lane1
10	Lane1Observed	Either values observed or imputed for lane1
11	Lane2TotalFlow	Number of vehicles in Lane2
12	Lane2AvgOcc	Average occupancy calculated w.r.t. lane2
13	Lane2AvgSpeed	Average speed calculated at lane2
14	Lane2Observed	Either values observed or imputed for lane2
.	.	.
.	.	.
.	.	.
35	Lane8TotalFlow	Number of vehicles in Lane8
36	Lane8AvgOcc	Average occupancy calculated w.r.t. lane8
37	Lane8AvgSpeed	Average speed calculated at lane8
38	Lane8Observed	Either values observed or imputed for lane8

**Table 2:** Traffic flow: The dataset schema used as input to our DL Model.

No.	Attribute Name	Description
1	stationId	This attribute defines the numeric value assigned to each vehicle detector station (VDS) on the highway. VDSs in PeMS are the data collection points on highways. Data used in this experiment include I5-N highway and has 26 VDSs.
2	dayOfMonth	Defines the day of a month in a Gregorian calendar in “dd” format. Thus, its value ranges from 1 to 31. This could be helpful in getting some traffic flow trends on some specific events, e.g traffic flow on Christmas every year.
3	month	Gives the numeric value for a Gregorian month in “mm” format and its value ranges from 1 to 12.
4	year	Value for a Gregorian year in the “yyyy” format.
5	hours	Clock hours in numbers starting from 0 to 23. This could help us identifying traffic flow trends and other information in specific hours in a day, e.g., traffic flow at 9 a.m.
6	weekDays	Day of a week, e.g., Monday. weekDays values are also in numeric format ranging from 1 to 7 where 1 represents Sunday, and 7 represents Saturday. This is important in identifying specific trends on specific days, e.g., on weekends.
7–18	flow (flow_00, flow_05, flow_10, …, flow_55)	As defined on PeMS, flow defines the number of vehicles passing through a vehicle detector station (VDS). In this dataset, we used five-minute interval flow values where the flow_00 defines the aggregated vehicle flow calculated at a VDS during the first five minutes of an hour. Similarly, flow_55 defines the aggregated flow value at a VDS during the last five minutes of the hour.

**Table 3:** Traffic flow: Deep model configuration.

No.	Description	Values
1	Number of input parameters	17
2	Number of output parameters	1
3	Number of hidden layers	4
4	Hidden units in each layer resp.	85, 425, 425, 85
5	Batch size	500, 5000
6	Number of epochs	100, 500, 1000
7	Activation function	ReLU

**Table 4:** Traffic speed: Deep model configuration.

No.	Description	Values
1	Number of input parameters	17
2	Number of output parameters	1
3	Number of hidden layers	5
4	Hidden units in each layer resp.	17, 85, 425, 425, 85
5	Batch size	1000, 5000, 10,000
6	Number of epochs	100, 200
7	Activation function	ReLU

**Table 5:** Traffic occupancy: Deep model configuration.

No.	Description	Values
1	Number of input parameters	17
2	Number of output parameters	1
3	Number of hidden layers	5
4	Hidden units in each layer resp.	17, 85, 425, 425, 85
5	Batch size	1000, 5000, 10000
6	Number of epochs	100, 200
7	Activation function	ReLU

**Table 6:** Comparison of traffic prediction approaches.

No.	Reference	Method	Forecast Parameter	Input Dataset	Dataset Size	Data Duration	Minimum MAPE
1	[26]	LSTM	Flow	Beijing Traffic Management Bureau	500 observation stations, 26 million records	6 months	6.05
2	[23]	Auto- encoders	Flow	PeMS	For all the highways in the system (GBs)	3 months	6.75
3	[27]	CNN, LSTM	Flow	PeMS	Includes 60 VDSs	2 months	NA
4	[84]	DBNs	Flow	PeMS	Roads with top 50 traffic flow values	12 months	9
5	[30]	LSTM	Speed	Self deployed detectors	Two sensors, 42387 records	1 month	3.78
6	This paper	LSTM	Speed	PeMS	Dataset used in our model	11 years	3.5
7	This paper	SVM	Speed	PeMS	Dataset used in our model	11 years	4.6
8	Our Model	CNN	Speed	PeMS	Dataset used in our model	11 years	2.59
9	Our Model	CNN	Flow	PeMS	Dataset used in our model	11 years	5.96
